# Replacing monocultures with mixed-species stands: Ecosystem service implications of two production forest alternatives in Sweden

**DOI:** 10.1007/s13280-015-0749-2

**Published:** 2016-01-07

**Authors:** Adam Felton, Urban Nilsson, Johan Sonesson, Annika M. Felton, Jean-Michel Roberge, Thomas Ranius, Martin Ahlström, Johan Bergh, Christer Björkman, Johanna Boberg, Lars Drössler, Nils Fahlvik, Peichen Gong, Emma Holmström, E. Carina H. Keskitalo, Maartje J. Klapwijk, Hjalmar Laudon, Tomas Lundmark, Mats Niklasson, Annika Nordin, Maria Pettersson, Jan Stenlid, Anna Sténs, Kristina Wallertz

**Affiliations:** Southern Swedish Forest Research Centre, SLU, Box 49, Rörsjöv 1, 230 53 Alnarp, Sweden; Skogforsk, Science Park, 751 83 Uppsala, Sweden; Department of Wildlife, Fish and Environmental Studies, SLU, 901 83 Umeå, Sweden; Department of Ecology, SLU, Box 7044, 750 07 Uppsala, Sweden; Department of Forestry and Wood Technology, Linnaeus University, 351 95 Växjö, Sweden; Department of Forest Mycology and Plant Pathology, SLU, Box 7026, 750 07 Uppsala, Sweden; Department of Forest Economics, SLU, Skogsmarksgränd, 901 83 Umeå, Sweden; Department of Geography and Economic History, Umeå University, 901 87 Umeå, Sweden; SLU, Skogsmarksgränd, 901 83 Umeå, Sweden; Foundation Nordens Ark, Åby Säteri, 456 93 Hunnebostrand, Sweden; Department of Business Administration, Technology and Social Sciences, Luleå University of Technology, 971 87 Luleå, Sweden; Department of Historical, Philosophical and Religious Studies, Umeå University, 901 87 Umeå, Sweden; Asa Research Station, SLU, 360 30 Lammhult, Sweden

**Keywords:** Climate change adaptation, Ecosystem services, Forestry, Mixed-forest stand, Polyculture, Resilience

## Abstract

Whereas there is evidence that mixed-species approaches to production forestry in general can provide positive outcomes relative to monocultures, it is less clear to what extent multiple benefits can be derived from specific mixed-species alternatives. To provide such insights requires evaluations of an encompassing suite of ecosystem services, biodiversity, and forest management considerations provided by specific mixtures and monocultures within a region. Here, we conduct such an assessment in Sweden by contrasting even-aged Norway spruce (*Piceaabies*)-dominated stands, with mixed-species stands of spruce and birch (*Betula pendula* or *B*. *pubescens*), or spruce and Scots pine (*Pinussylvestris*). By synthesizing the available evidence, we identify positive outcomes from mixtures including increased biodiversity, water quality, esthetic and recreational values, as well as reduced stand vulnerability to pest and pathogen damage. However, some uncertainties and risks were projected to increase, highlighting the importance of conducting comprehensive interdisciplinary evaluations when assessing the pros and cons of mixtures.

## Introduction

Ecosystem services refer to the benefits people obtain, either directly or indirectly, from ecosystems (Nahlik et al. 2012). Production forests provide a diverse range of ecosystem services beneficial to societal wellbeing, including for example the storage and sequestration of atmospheric carbon, wood for building and energy, and environments for recreation. Despite the breadth of this capacity, forest management models are often adopted which enhance the delivery of single services, such as timber, to the detriment of other services, such as regulatory or cultural services (Bennett et al. 2009; Raudsepp-Hearne et al. 2010). A key challenge this century is to identify production forest alternatives better suited to the sustainable provision of a breadth of such services for a growing human populace (Gustafsson et al. 2012).

Whereas monocultures have excelled at providing large quantities of wood per unit area, this has often come at the expense of biodiversity (Lindenmayer and Franklin 2002), with resultant implications for additional ecosystem services (Jactel et al. 2009; Griess and Knoke 2011). In contrast, mixed-species approaches to production forestry, in which stands are designed around the targeted production of two or more tree species, may be less prone to such stark tradeoffs, and may even provide increased production and economic outcomes relative to monocultures (Griess and Knoke 2011; Paquette and Messier 2011; Gamfeldt et al. 2013; Bielak et al. 2014). Furthermore, the risks, uncertainties and increasingly observed damage inflicted on production forests by climate change (Seidl et al. 2014), may favor the increased use of mixed-species stands, as they provide managers with alternative directions for future stand development (Millar et al. 2007).

Whereas there is evidence that tree species mixtures in general provide a breadth of potential benefits relative to monocultures (Gamfeldt et al. 2013), the extent to which multiple ecosystem services can be simultaneously derived from specific mixed-species alternatives is less clear. For many regions, it remains to be determined how well individual mixed-species alternatives can balance the net tradeoffs and synergies among ecosystem services and adaptive capacity. Providing relevant insights in this regard requires scientific evaluations of an encompassing suite of ecosystem services, biodiversity, and other considerations derived from specific mixture versus monoculture forestry alternatives, within a given biogeographical context. From such studies, insights can be gained regarding the collective benefits and tradeoffs of a given mixed-forest alternative, with outcomes of relevance to forest owners, managers, and policy makers. Such studies should provide a more justified basis for motivating the adoption of mixed-species approaches, or alternatively, a better understanding of the reasons behind the continued widespread reliance on monocultures (Kelty 2006).

Here we conduct such an assessment in Sweden, where current policies and environmental goals are actively supporting the adoption of mixtures (SOU 2013). Our reference condition consists of a subset of Sweden’s even-aged Norway spruce (*Piceaabies*; hereafter spruce)-dominated stands. We contrast this reference condition with two mixed-species production forest alternatives which dominate scientific consideration and the public discourse in Sweden: mixtures of spruce with either birch (*Betula pendula* or *B. pubescens*, hereafter birch) or Scots pine (*Pinussylvestris*; hereafter pine). We evaluate the incentives, obstacles, and implications from the combined perspectives of biodiversity conservation, silviculture, production, economics, recreation, esthetics, ecological risks, water quality, and adaptive capacity. Our primary aim is to provide an overview of a broad range of relevant considerations, rather than a comprehensive review of each topic assessed.

## Materials and methods

### Reference stand condition

Norway spruce is the most common tree species by volume on productive forest lands in Sweden, though its dominance is supplanted by Scots pine in the central and northern parts of the country (SFA 2014). Due to the high proportion of forest area in southern Sweden consisting of spruce-dominated production stands (~40 % of Götaland’s forest area; Drössler 2010), converting some of these stands to other tree species or mixtures is considered a means of reducing the susceptibility of the forest estate to climatic uncertainty and specific abiotic and biotic risks (Zhang and Schlyter 2004; Thor et al. 2005; Felton et al. 2010a; Valinger and Fridman 2011). During early stages of spruce stand development, other naturally regenerating tree species can represent a substantial proportion of volume, but most of these tree species are generally removed by thinning during the first half of the rotation. To increase the percentage of broadleaf tree species, the Forest Stewardship Council (FSC) now requires the retention of ≥10 % broadleaved tree species by volume (5 % in the north of the country) (FSC 2010). Using this requirement as a guideline, the reference condition for our assessment consists of stands designed specifically for the production of spruce, and managed so that spruce comprises ≥90 % of stand volume during the second half of the rotation. This stand type is the most common category of production forest in southern Sweden (Götaland), representing over 20 % of total forest area (Drössler 2010). We refer to these stands as spruce monocultures.

### Mixture alternatives

We contrast this reference stand condition with two mixed-species alternatives. Definitions of mixtures vary extensively (see Bravo-Oviedo et al. 2014), but for our purposes we define mixtures as stands designed around the simultaneous production of two tree species, mixed stem wise, each of which comprising ≥30 % of stand basal area at the time of final harvest. The first mixture alternative assessed comprises planted spruce and naturally regenerated birch. The second alternative involves mixtures of planted spruce and naturally regenerated pine. The mixed-species production alternatives considered represent two of the most common conifer–broadleaf mixtures and purely conifer mixtures in southern Sweden (Drössler 2010).

### Synthesis methods

Researchers with expertise in a range of relevant disciplines summarized the current state of scientific evidence regarding the implications of these stand types for biodiversity, and a select set of provisioning (wood production and water), cultural (recreation and esthetics), and regulatory services (reduced risks of pests, pathogens, fire, windthrow, and browsing damage). The choice of topics assessed was limited by the expertise of participating researchers, and thus to some extent subjective. The spatial grain of interest was the stand rather than landscape level. Whereas some of the topics covered address early stages in the rotation (e.g., regeneration), most relate to the second half of the rotation unless otherwise specified. We also summarize additional forest management considerations which are likely to be of importance to decision makers, but which do not fit within the other categories considered. These include adaptive capacity, financial security, regeneration, logging costs, and management simplicity.

**Fig. 1:**
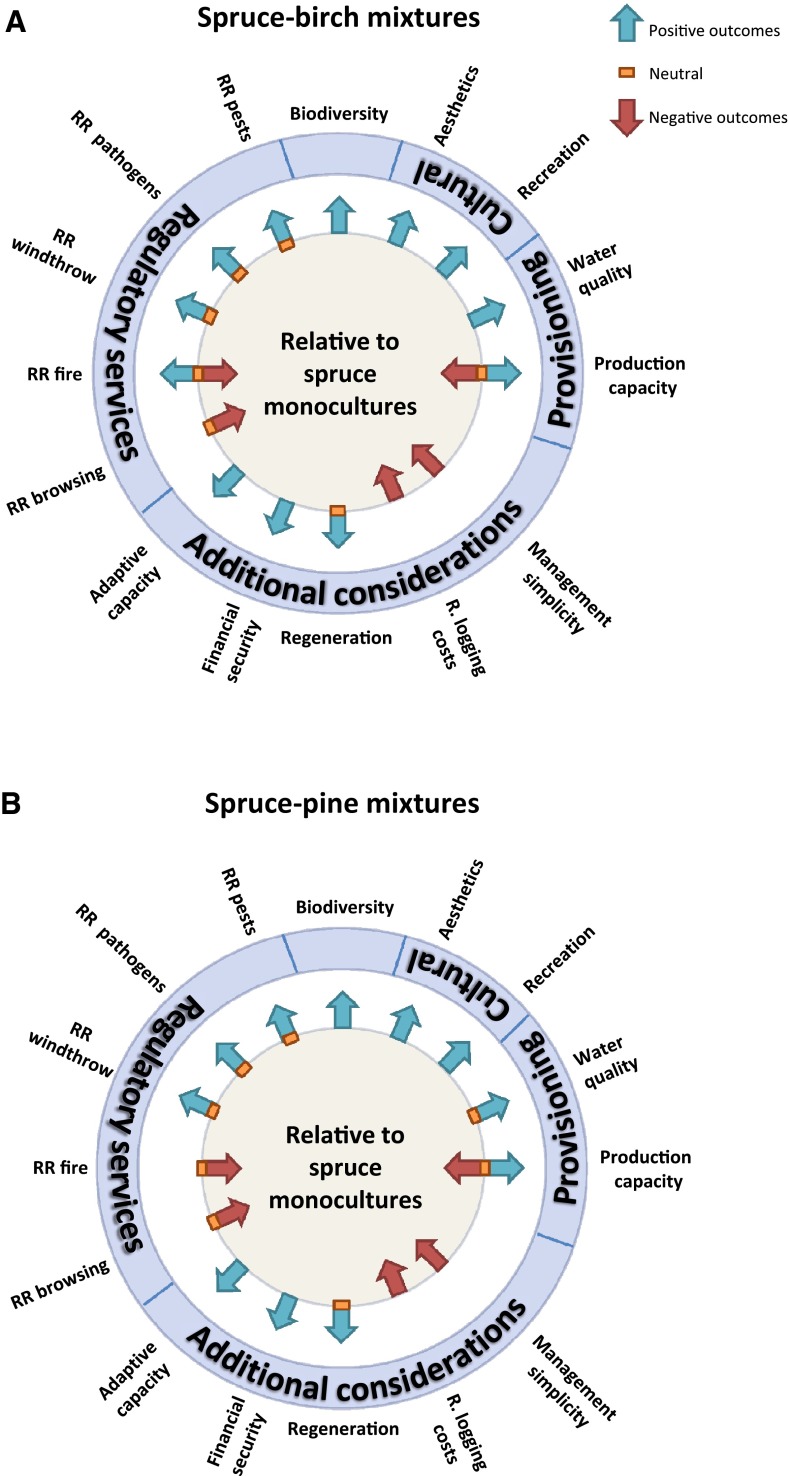
Summary diagrams illustrating positive, neutral, or negative outcomes of **a** spruce–birch and **b** spruce–pine mixtures relative to spruce monocultures in terms of biodiversity, provision, regulatory (RR = reduced risk), and cultural services, as well as additional considerations of likely relevance to forest owners and managers. The direction, or neutrality, of the arrow is used to indicate where the weight of currently available scientific evidence falls, as summarized in the accompanying text. In those circumstances where such a conclusion could not justifiably be reached, we use a combination of positive, neutral, or negative indicators to acknowledge the extent of uncertainty. The figure is designed so that positive outcomes for biodiversity, ecosystem services, and additional considerations increase outwards relative to the central spruce monoculture reference condition. See accompanying text for further details and caveats. We include hunting and the collection of non-wood forest production under recreational activities, despite their relevance to provisioning services

As part of this synthesis, electronic databases were searched using different combinations of Boolean search terms to capture the relevant scientific literature. The databases used were Google, Google Scholar, and Web of Science. A core set of search terms, “*Picea abies*” and (“*Pinus sylvestris*” or “*Betula*”) and (mix* or polyculture* or admix*), was used by participants, and supplemented with additional search terms targeted to capture those studies of relevance to specific topics of interest (e.g., “pest*,” “pathogen*,” “biodivers*”). Search terms were run in separate or limited combinations depending on the requirements or limitations of the database used. We also obtained results from colleagues, books, and government reports, and from the reference lists of published studies. Due to the range of issues we attempt to address, as well as space and citation limitations, the results provided are best seen as a condensed overview. In order to convey these results in a readily digestible format, we have simplified outcomes further in a summarizing illustration of the collective results for each mixture assessed (Fig. 1a, b). As the topics chosen for inclusion, as well as the boundary delineation for each topic, are to some extent subjective, the outcomes illustrated are intended as an overview of our findings, rather than as a basis for quantifying the entirety of potential costs and benefits derived from each stand type. See figure legend for more details.

## Results

### Climate change adaptation

For moderate-to-high emission scenarios of greenhouse gases over the coming century, model projections indicate mean annual temperature increases of 2–7 °C by 2071–2100 for Sweden, compared with the reference period 1961–1990 (Kjellström et al. 2014). Northern Sweden will face the largest increases in temperature, and temperature increases will be larger in winter (2–9 °C) than summer (1–6 °C). Precipitation is projected to increase by up to 40 %, but with large variations between years and decades. Notably, some projections indicate decreased precipitation during summer for southern Sweden, of potential detriment to spruce. In terms of growing conditions, broadleaf tree species in general are expected to benefit from climate change in southern Sweden (Lindner et al. 2014). More specifically, some projections indicate that all three tree species considered will benefit under moderate-to-high GHG emission scenarios, with pine experiencing the highest relative increases in net primary production relative to both birch and spruce (Bergh et al. 2010). However, large uncertainties regarding the associated growth benefits of elevated CO_2_ cause projections to vary from neutral to positive in terms of tree species growth rates over the coming century (Lindner et al. 2014). In addition, the potential adverse implications for spruce in southern Sweden due to elevated risks posed by windthrow, bark beetle outbreaks, spring frosts, and summer droughts (Grundmann et al. 2011) need also to be taken into consideration. In summary, the use of both mixture alternatives may be favored in southern Sweden due to their inclusion of tree species projected to benefit under climate change, noted abiotic and biotic threats to spruce, and the increased adaptive capacity provided for by mixtures (Fig. 1).

### Biodiversity

Converting spruce monocultures to spruce–birch or spruce–pine mixtures will increase the range of environmental conditions provided, and therefore the variety of potential habitats found within the stand. This is especially the case for spruce–birch mixtures, as the phylogenetic distinctiveness between these tree species favors the establishment of flora and fauna specifically evolved to exploit either the mixture *per se* (Jansson and Andren 2003) or each tree species’ characteristic resources and structures (Jonsell et al. 1998). In the case of spruce–birch mixtures, the addition of a broadleaf tree species will likely increase levels of soil insolation and rates of nutrient cycling, raise soil quality in terms of mineral content and carbon:nitrogen ratio, and therefore benefit the diversity of vascular plants and associated taxa (Barbier et al. 2008). A systematic review specifically assessing spruce monocultures versus spruce–birch mixtures in Sweden concluded that stand-scale increases in species richness and abundance may be expected for birds, understory vegetation, saproxylic beetles, and lichens (see Felton et al. 2010b). However, improved micro-climatic conditions for understory herbaceous vegetation may come at the expense of ground-living bryophyte diversity and abundance. Some conifer specialists could also experience a decrease in habitat (but see Felton et al. 2011).

In contrast to spruce and birch, spruce and pine are of relatively closer physiognomy and phylogenetic relatedness. This could decrease the benefits for biodiversity from mixing these tree species. However, a positive effect is nevertheless likely due to the extent of difference between pine and spruce in terms of their respective bark and dead wood characteristics, and the resultant micro-climatic and soil conditions that arise from their distinctive crowns, branches, and needles (Kuusinen 1996; Jonsell et al. 1998; Barbier et al. 2008). Furthermore, differences in species diversity and composition between spruce and pine stands, or for individual trees, have been identified in a number of European studies. For example, the species composition (and vertical stratification) of epiphytic lichens found on mature pine and spruce trees differs (Marmor et al. 2013); macrofungi communities in planted pine and spruce stands contain many species unique to either of the stand types (Ferris et al. 2000); spruce stands can contain a higher diversity of bryophytes than pine stands (Augusto et al. 2003); and mixtures of spruce and pine can contain higher bird species diversity, and distinctive bird species composition, than monocultures of spruce (Gjerde and Saetersdal 1997).

Increased biological diversity can thus be expected if spruce monocultures are converted to either spruce–birch or spruce–pine mixtures (Fig. 1a, b). However, biodiversity outcomes will depend on a variety of variables, including stand proximity to source populations, the relative proportions and juxtaposition of tree species, the extent to which management regimes allow understory vegetation to develop, and the extent of conservation measures implemented (e.g., green tree and dead wood retention).

### Regulatory services

#### Windthrow

The risk of windthrow depends on the tree species considered, stand exposure, tree height, stem density, time since thinning, and the season (Griess et al. 2012). Whereas climate projections for Sweden provide no clear indications of changes to wind intensities, or the frequency of high-wind events (Kjellström et al. 2014), projected milder and wetter winters with less soil freezing make trees more conducive to windthrow. In Sweden, damage associated with high-wind events has increased over recent decades (Schlyter et al. 2006), and spruce is considered to be particularly susceptible in this regard (Valinger and Fridman 2011). Mixing spruce with tree species such as birch or pine, which are considered to have a higher mechanical stability (Peltola et al. 2000), could improve the overall wind stability of stands otherwise dominated by spruce (Dhôte 2005). A study of stand susceptibility to wind damage (defined by wind damage to a single tree or more) after a major storm indicates that the risk to spruce from storm felling decreased by over 50 % when grown in stands with 30 % broadleaf trees in general, or birch specifically (Valinger and Fridman 2011). Reduced windthrow was also observed, but to a lesser extent, from the addition of pine. However, caution is always warranted when extrapolating from the results of single disturbance events (Valinger and Fridman 2011). Furthermore, it is also possible that the site conditions which favor the addition of other tree species within a stand also help reduce windthrow. Nevertheless, these findings are consistent with study results elsewhere in Europe which likewise highlight how a higher proportion of spruce in a stand can increase the risk of windthrow (Griess et al. 2012).

#### Fire

For most climate change scenarios, a drier summer climate is projected for some regions of southern Sweden later this century. If coupled with prolonged periods without precipitation, this may increase the risk of forest fires (Kjellström et al. 2014). The relative vulnerability of a particular forest stand to fire will however depend on the availability of fuel, its distribution within the stand (e.g., ground fuels or crown fuels), and flammability (Schelhaas et al. 2010). All of these aspects are strongly influenced by tree species composition (Jactel et al. 2009). In general, conifer foliage is more flammable than broadleaf trees due to the higher content of resins and oils (Bond and van Wilgen 1996), with corresponding implications for ground fuels. For this reason, fire risk in mixtures with broadleaves is usually lower than in pure conifer stands (Gonzalez et al. 2006). However, if the density of a pure spruce stand is sufficiently high, the environments created can greatly limit the flammability of ground fuel. Relative to such stands, fire risk could theoretically increase in spruce–birch mixtures, depending on the extent to which increased light levels promote understorey vegetation and associated fuel loads, and whether fallen birch leaves act to suppress or enhance the flammability of the understorey (Berglund 1998). Rainfall, temperature, site conditions, and stand structure are all important determinants in this regard (Fig. 1a). In contrast, spruce–pine mixtures will likely increase the fire hazard relative to spruce–birch mixtures or spruce monocultures (Fig. 1b), due to the lower fuel moisture and higher ignition potentials associated with pine trees and the stand conditions they promote (Tanskanen et al. 2005).

#### Pests and pathogens

Relative to monocultures, the use of mixed-species stands may be expected to reduce the risk of pest and pathogen outbreaks (Pautasso et al. 2005; Jactel et al. 2009), as negative correlations between tree species diversity and the level of damage from such organisms are often identified (Jactel and Brockerhoff 2007). Several potential mechanisms have been proposed to account for these results, based on both the prevalence of host trees and the diversity of predators and parasitoids of pest species (Tahvanainen and Root 1972; Root 1973). Reduction in the proportion of susceptible trees or the proximity/abundance of non-host plant species could, for example, decrease host tree detection or transmission potential (Keesing et al. 2006; Barbosa et al. 2009). Alternatively, increased tree diversity can have a direct or indirect positive effect on the abundance and diversity of the natural enemies of pest species (Underwood et al. 2014).

In Sweden, the use of mixtures may be able to reduce the risk of damage by the most destructive pathogen affecting spruce, *Heterobasidion annosum* sl., as indicated by modeling studies (Thor et al. 2005). Although the results of *H. annosum* studies do vary, damage from *H. annosum* is often reduced when spruce is mixed with pine, but less evidence is provided for spruce–birch mixtures (Korhonen et al. 1998). Transmission rates of other pathogens of spruce, such as *Armillaria* spp., also appear to be reduced with increasing tree diversity (Gerlach et al. 1997), and a reduced proportion of spruce within a stand can also limit the colonization of needles by endophytic fungi (Muller and Hallaksela 1998).

With respect to insect pests, one of the most damaging to spruce is the spruce bark beetle *Ips typographus*. The risk of spruce bark beetle damage in a stand is often lower when the proportion of spruce in a stand is reduced (Overbeck and Schmidt 2012), likely due to associated reductions in the population densities of this pest species. Spruce bark beetle damage can also be lowered by adding birch to a stand, as the volatiles from these non-host tree species can help deter spruce bark beetles (Zhang and Schlyter 2004), a result which mirrors studies of other insect herbivores (Jactel et al. 2011). The pine weevil *Hylobius abietis* is also of substantial concern in spruce stands, as this insect pest causes the most damage to conifer seedlings. Whereas this pest species is expected to decrease in spruce stands with an increasing proportion of birch (Björkman et al. 2015), the addition of pine cannot be expected to provide similar benefits. However, pest outbreaks may also be reduced by increasing the presence of their predators or parasitoids (Jactel et al. 2009). In this regard, the addition of pine to a stand may reduce damage by increasing the abundance of predatory ants (Koricheva et al. 2006). Likewise, the abundance of pest-controlling species has been found to increase in spruce–birch mixtures relative to spruce monocultures (Vehviläinen et al. 2008), and relatedly pest damage to birch is also found to decrease with an increasing percentage of spruce in a stand (Vehviläinen et al. 2007).

Assessing the potential for mixtures to reduce the risk of pest and pathogen damage involves a number of additional considerations. First, the extra tree species may itself be vulnerable to pests or pathogens at a given site. For example, the potential for rust fungus *Melampsoridium betulinum* outbreaks must be considered when using birch, whereas pine trees can be infected by the shoot fungus *Gremmeniella abietina*. Furthermore, whereas damage by specialist pest and pathogen species can be reduced in mixtures, damage by generalists species may instead increase (Plath et al. 2012). Finally, outcomes are also dependent on the specific nature and context of the mixture (Vehviläinen et al. 2007; Castagneyrol et al. 2013). Therefore, even though the current weight of evidence indicates reduced damage in mixtures for many of the better known pest and pathogen species found in these stands (Fig. 1), large uncertainties remain, particularly with respect to the responses of other less-studied pest and pathogens.

#### Ungulate browsing

In Sweden, local population densities of large browsing herbivores can be high. These species, including moose (*Alces alces*) and roe deer (*Capreolus capreolus*), often browse on the bark, twigs, and foliage of production tree species. The use of spruce–birch or spruce–pine mixtures raises concerns regarding increased browsing damage (Fig. 1), because ungulates will generally prefer to browse on pine and birch than spruce (Månsson et al. 2007). Furthermore, browsing impacts have been observed to increase in mixtures when birch or pine is present (Vehviläinen and Koricheva 2006; Milligan and Koricheva 2013), with associated increases in browsing pressure on the spruce found within such stands (Milligan and Koricheva 2013). How much a particular stand is affected by browsing will however vary depending on a range of factors, such as local ungulate densities and the availability and quality of alternative sources of forage (Månsson et al. 2012). In either regard, the addition of two tree species generally preferred as forage by large ungulates has the potential to increase the risk of browsing damage in individual production forest stands under current circumstances.

### Cultural services

#### Esthetics and outdoor recreation

Outdoor recreation is an important national tradition in Sweden (Fredman et al. 2014), and forests are regularly used for such activities. The most common recreational activities in forests include social visits (e.g., picnics with family and friends), the pleasure of nature experiences, physical activities (e.g., walking, running, biking, and skiing), and the hunting or collection of forest products (e.g., game meat, berries, mushrooms, or herbs) (Lisberg Jensen and Ouis 2014). Different recreational activities will favor different kinds of forest settings, and recreational preferences for mixtures versus monocultures have only been studied in limited detail, yielding somewhat contradictory outcomes. However, in general, surveys indicate that variation in forest color and texture provided by distinctive tree species is often preferred esthetically (Olsson 2014). Relatedly, mixtures are often preferred esthetically over monocultures (Gundersen and Frivold 2008), and this preference is sometimes linked to resultant increases in understorey light levels and openness (Eriksson et al. 2012). The use of broadleaved trees and pine is thus considered favorable in increasing the esthetic value of a stand (Fig. 1), especially in production forests located close to residential and recreation areas.

Berry collection is a strong motivator for forest visits. Bilberry (*Vaccinium myrtillus*) is one of the most economically important wild berry species in Sweden and is widely collected for both household consumption and sale (Lindhagen and Bladh 2013; Sténs and Sandström 2013). Bilberry is more common in plots with multiple tree species than in monocultures of spruce (Gamfeldt et al. 2013), and its occurrence is specifically associated with pine (Miina et al. 2010). The collection of edible mushrooms is also important in Sweden, with such collections estimated to exceed 15 million liters in some years (Yrjölä 2002). The most popularly consumed mushrooms in Fennoscandia have mycorrhizal associations with pine, spruce, and/or birch, though these associations show varying degrees of host specificity (Salo 1995). Whereas the conversion of spruce monocultures to spruce–birch and spruce–pine mixtures is thus likely to influence the occurrence and production of edible mushrooms, there are insufficient studies to confidently project their likely response (Pilz and Molina 2002; Pinna et al. 2010; Savoie and Largeteau 2011).

Game animals are an important hunted resource in Sweden, for which the annual gross value of recreational benefits and the food provided is estimated to be over 300 million USD (Boman and Mattsson 2012), with additional value provided to non-hunters and tourists. Moose and roe deer are some of the most economically important game species, for which young pine and birch constitute a substantial part of their winter diet (Cederlund et al. 1980). Spruce–birch or spruce–pine mixtures will increase the availability of winter forage within landscapes, thereby favoring their populations. Spruce–pine mixtures also benefit populations of important game birds, such as capercaillie (*Tetrao urogallus*) (Summers et al. 2004), whereas spruce–birch mixtures provide valued habitat for hazel grouse (*Tetrastes bonasia*) (Åberg et al. 2003). The addition of pine or birch to otherwise spruce-dominated stands should therefore also increase the hunting-related recreational value of these stands (Fig. 1).

### Provisioning services

#### Water quality

In riparian zones, tree species composition is of direct relevance to ecosystem processes in streams (Kuglerová et al. 2014). In boreal forests, riparian stands, which include a higher component of broadleaf trees, appear to improve stream conditions by varying levels of insolation and increasing the amount of leaf litter (Burrows et al. 2015). Light and organic litter input is in turn associated with the development of heterotrophic biofilms (Hill et al. 2009), which play a fundamental role in the retention of stream nutrients and support the occurrence of higher trophic-level aquatic organisms (McKie and Malmqvist 2009). The inclusion of broadleaves into otherwise conifer-dominated riparian stands may therefore help reduce concentrations of inorganic nitrogen leaching to streams (Gundersen et al. 2006). Compared to broadleaf litter, conifer needles decompose more slowly and hence produce higher soil concentrations of dissolved organic carbon (DOC) (Camino-Serrano et al. 2014), which subsequently lead to higher export from conifer-dominated stands (Grabs et al. 2012). Referred to as “brownification,” DOC has numerous negative effects on water quality and delays the capacity of streams to recover from acidification (Erlandsson et al. 2011). For these reasons, the use of spruce–birch mixtures rather than spruce monocultures should contribute to improved aquatic environments and downstream water quality. Stands that comprised spruce–pine mixtures could also be expected to diversify stream insolation levels and therefore improve habitat quality; however, any potential benefits to water quality are unlikely to be commonplace due to pine’s rare association with riparian zones (Fig. 1).

#### Wood production

**Fig. 2:**
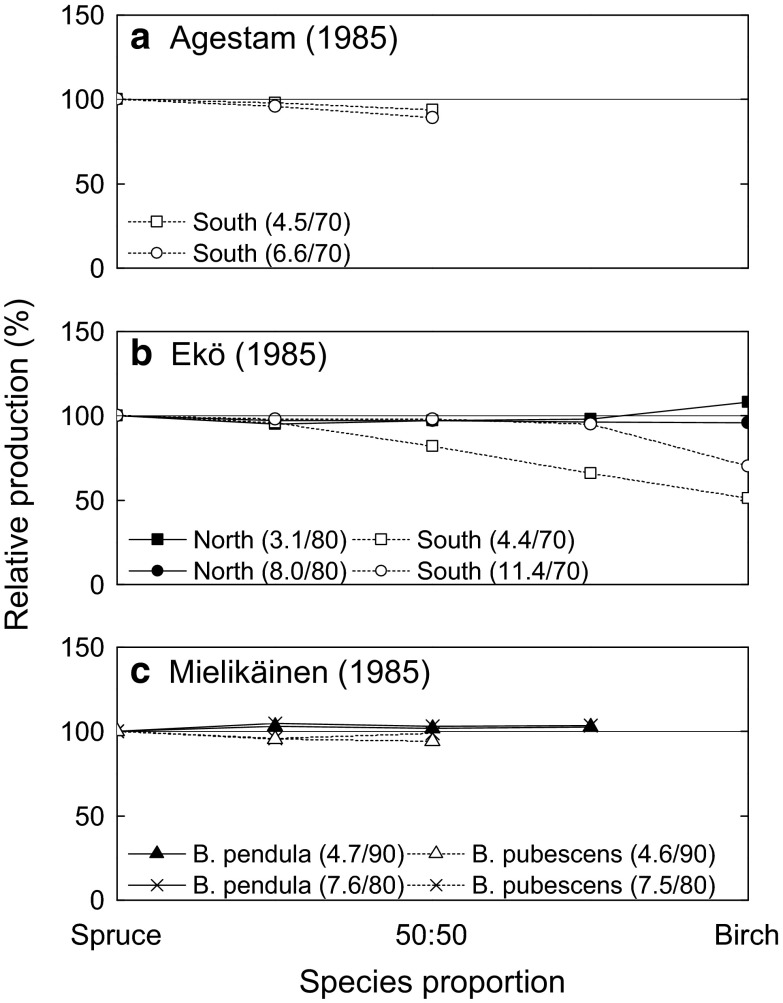
Relative stem volume production for mixtures and pure stands of Norway spruce and birch from the few simulations which consider the whole rotation, by **a** Agestam (1985) and **b** Ekö (1985) for Sweden and **c** Mielikäinen (1985) for Finland. Relative production (%) is presented in relation to pure spruce stands. Separate simulations are presented for the northern and southern parts of Sweden (**a**, **b**) and for different site fertility classes (MAI (m^3^ ha^−1^ year^−1^) for pure spruce and rotation length (years) within brackets). Mielikäinen (1985) also distinguished between birch species

The wood production capacity of mixtures may exceed that of monocultures, if (a) complementary resource exploitation leads to more complete use of environmental resources, (b) the additional species modifies the environment in a way which facilitates the growth of a co-occurring species, or (c) the stand-level response to disturbance is less intense and provides faster recovery times (Fridley 2001; Kelty 2006; Jactel et al. 2009). Unfortunately, there are few published studies contrasting either spruce–birch or spruce–pine mixtures with spruce monocultures on similar sites within northern Europe. Instead, what is available are a handful of relevant studies using a variety of approaches which provide insufficiently consistent results to draw firm conclusions. For example, a correlative study built on Sweden’s National Forest Inventory data found a positive relationship between biomass production and the number of tree species in sample plots (Gamfeldt et al. 2013). However, interpreting yield comparisons based on correlative findings can be problematic, as a plot containing a higher diversity of tree species is also more likely to contain tree species with a high productive capacity within a given site (Fridley 2001; Bravo-Oviedo et al. 2014). This so-called “sampling effect” is difficult to eliminate statistically. Furthermore, such study results often diverge from those provided by experiments and other methodologies (Drössler et al. 2015). For example, simulations using growth models based on data from large nation-wide inventories, and representing a wide range of site and stand types, indicate similar or lower yields in spruce–birch mixtures than spruce monocultures throughout a rotation (Fig. 2; Agestam 1985; Ekö 1985). However, caution is also warranted when interpreting these results, as modeling studies based on measurements in randomly selected forest plots, such as those depicted in Fig. 2, may underestimate birch growth rates, as birch presence can be indicative of lower management ambition (Mielikäinen 1985). Furthermore, the common approach of pooling the two birch species in comparative studies can obscure the volume production capacity of either birch species under different site conditions (Fig. 2c, Mielikäinen 1985).

Interpreting the results of empirical studies is also complicated. Field studies from Norway indicate that up until 17 m in height, spruce–birch mixtures yield more than spruce monocultures, whereas no significant differences were found at greater heights (Frivold and Frank 2002). Several studies have also found that leaving a shelter of young birch over spruce during the first part of the rotation can increase total production (Tham 1988, 1994). However, studies on fertile sites in southern Sweden indicate no significant difference in yields during the 10 years following pre-commercial thinning in single-storied mixtures (Fahlvik et al. 2011). Outcomes can also vary depending on the metric assessed. If dry weight rather than stem volume is assessed, spruce–birch mixtures can provide more favorable production outcomes than spruce monocultures, particularly in relation to bioenergy production (Mielikäinen 1985). To summarize, projecting production outcomes for spruce–birch versus spruce monocultures remains ambiguous, and will likely vary depending on the site conditions, the wood product desired, the birch species assessed, and the time period during the rotation considered (Fig. 1a).

**Fig. 3:**
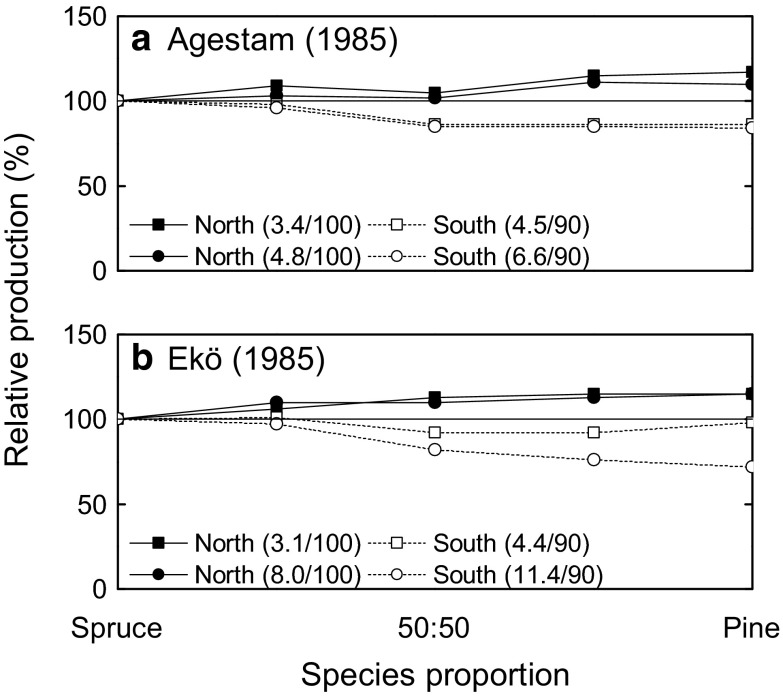
Relative stem volume production for mixtures and pure stands of Norway spruce and Scots pine from simulations which consider the whole rotation, by **a** Agestam (1985) and **b** Ekö (1985). Relative production (%) is presented in relation to pure spruce. The figure shows separate simulations for the northern and southern parts of Sweden (**a**, **b**) and for different site fertility classes (MAI (m^3^ ha^−1^ year^−1^) for pure spruce and rotation length (years) within brackets)

Studies assessing the wood volumes produced by spruce–pine mixtures versus spruce monocultures also provide inconsistent outcomes (Fig. 1b). Simulations based on repeated measurements of randomly selected forest plots indicate that as the proportion of pine is increased, similar or higher production outcomes may occur for mixtures in the north, whereas lower yields are indicated in the south (Fig. 3, Agestam 1985; Ekö 1985). In contrast, field studies from central Sweden indicate that spruce–pine mixtures can in fact provide higher yields than spruce monocultures during the middle of the rotation. Notably, these results appear to be driven primarily by pine’s higher production capacity for these site conditions, rather than indicating a mixture benefit per se (Jonsson 2001). In southern Sweden, assessments find no significant differences in volume increment between pure Norway spruce and spruce–pine mixtures during the first half of the rotation (Lindén and Agestam 2003).

Tree species interactions can lead to “over-yielding” in which higher production outcomes are achieved in mixtures, relative to the average of two comparable sized pure stands that comprised the component tree species (Pretzsch and Schütze 2009). Whereas over-yielding may be occurring in some spruce–pine and spruce–birch mixtures in Sweden, continuing uncertainties highlights the need for targeted long-term experiments.

### Additional considerations

#### Regeneration

In general, levels of natural regeneration by birch and pine present an opportunity rather than an obstacle to mixed-species stands. Recent assessments indicate that over 50 % of clear cuts already rely on natural regeneration to reach legislative requirements for stocking densities (Bergquist et al. 2011). Furthermore, pre-commercial thinning is often necessary to remove the undesired excess of natural regeneration. In addition, scarification methods which favor natural regeneration are already standard practice. As such, if spruce–birch or spruce–pine mixtures are desired, regeneration costs could be reduced by lowering the density of planted spruce seedlings and, by so doing, further increase opportunities for the natural regeneration of tree species in the subsequent pre-commercial thinning (Holmström et al. 2015). Nevertheless, site conditions need to be taken into consideration when determining suitable alternatives for stand development, and large annual variation in seed production and establishment can limit successful regeneration in some years (Karlsson 2001).

#### Management simplicity

Compared to even-aged monocultures, the management of tree species mixtures requires additional management considerations. Tree species possess distinctive ecological traits, which allows for complex interactions and feedbacks within mixtures, depending on site conditions, the period in the rotation, and management interventions (Pretzsch and Schütze 2009; Forrester 2014). In practice, specific silvicultural treatments developed for monocultures should be adapted to accommodate the use of two or more tree species, rather than being optimized for one. However, silvicultural recommendations in Sweden, as in many countries, are largely based on knowledge derived from monocultures, either through the use of field experiments or practical experience. For example, of the more than 1600 long-term silvicultural experiments taking place in Sweden, approximately 3 % are conducted in mixtures (www.silvaboreal.com). Due to the resultant lack of local knowledge regarding suitable silvicultural prescriptions, the tree species found in mixtures are often managed using recommendations designed for monocultures. For these reasons, the complexities and uncertainties of using spruce–birch and spruce–pine exceed those associated with spruce monocultures at present (Fig. 1). These obstacles could however be readily overcome by a sustained shift in research funding toward studies of mixed-species forests.

#### Logging *costs*

Logging costs primarily depend on the methods of extraction used, the stage at which the harvesting takes place (thinning or final felling), and the size of trees being logged. An important determinant of such costs is the need for “assortments,” which refers to the separation of timber into piles for transport, based on their size, quality, or species. The species of tree generally harvested in Scandinavia (spruce, pine, birch) has only insignificant implications for logging costs in the mechanized harvester–forwarder systems used (Kuitto et al. 1994) and, in forestry, is generally neglected as a financial consideration. In contrast, costs tend to increase with the number of timber assortments, due to associated increases in harvesting and transportation costs from the stump to a roadside landing (forwarding). Increased costs per additional assortment are approx. 1 % during harvesting and 3–4 % during forwarding (Brunberg and Arlinger 2001). These costs are considered to be either similar for both the thinning and final felling operations, or alternatively higher during thinning operations (3 %) than final felling (1–2 %) (Sirén and Aaltio 2003). Relative to spruce monocultures, the number of assortments in spruce–birch mixtures typically increases the costs during the first thinning by 2 %, with a 4 % increase for both the second thinning and final felling. The number of assortments in spruce–pine mixtures increases logging costs during thinning by 0–2 %, and by 0–6 % at final felling. Logging costs can therefore be expected to increase with the use of mixtures (Fig. 1).

#### Financial security

Projecting the economic returns from timber production requires consideration of both expected value and variance (Andersson and Gong 2010). When conducting such projections for mixtures and monocultures, timber price fluctuations (Hultkrantz et al. 2014) and difficulties in projecting timber yield lead to high levels of uncertainty. As a result, and due to a number of additional context-specific considerations, the value of economic returns from spruce–birch or spruce–pine mixtures may be higher, lower, or equal to that provided by spruce monocultures.

Nevertheless, because timber prices for different tree species are not perfectly correlated, the economic returns from mixtures tend to be less sensitive to variations in timber prices than monocultures. This reduces the variance in economic returns and, as a result, lowers financial risk (Knoke et al. 2005). Mixtures also enable owners to adapt their commercial thinning regimes in response to realized price differences for the component tree species (Lu and Gong 2005). This flexibility also extends to decisions regarding which of the two tree species should comprise more or less of the stand’s volume, with associated implications for the income derived at final harvest. Furthermore, and as noted above, the use of mixtures can reduce some ecological risks and therefore further reduce uncertainty in timber yield. For these reasons, mixed-species stand can be expected to provide better financial security than a monoculture.

## Discussion

Relative to spruce monocultures, the adoption of spruce–birch or spruce–pine mixtures in Sweden can be expected to produce positive outcomes for forest biodiversity, water quality, and esthetic and recreational values, as well as likely reducing stand vulnerability to pest and pathogen damage (Fig. 1). These results support the contention that specific tree species mixtures can in fact achieve many of the broad categories of benefits commonly associated with mixtures in general. If any of these specific outcomes are prioritized over other considerations, then spruce–birch and spruce–pine mixtures appear to be clearly preferable production forest alternatives to spruce monocultures. In general, however, such results must be considered as part of the complex suite of incentives and disincentives for adopting mixtures, for which each decision maker will likely vary in how they prioritize any single concern, uncertainty, or benefit (Puettmann et al. 2015). Our results also highlight that even within targeted categories of concern, such as provisioning or regulatory ecosystem services, the emergent picture was complex. Whereas the two mixtures considered did reduce some stand vulnerabilities, other risks were projected to increase. Likewise, though some production and economic outcomes were likely to improve, other costs would be incurred. Overall, both mixtures considered were deemed to result in positive outcomes for the majority of issues assessed, but the conclusions reached from our assessment will nevertheless be dependent on the values that stakeholders place on the different ecosystem goods and services.

With respect to the potential wood production capacity of these mixtures, there are too few experimental studies to draw definitive conclusions for the variety of Swedish conditions. It is important to note however that even in those circumstances where equal or higher production capacity could safely be projected for particular site conditions, this may not result in mixture adoption. Previous studies emphasize that owners and managers frequently lack the necessary confidence and knowledge to switch to mixtures, despite proven production benefits. This reluctance is often linked to the associated increase in management complexity and related uncertainties regarding outcomes (Knoke et al. 2008; Pawson et al. 2013; Puettmann et al. 2015).

Economic outcomes are also context dependent, varying for example with harvesting costs, species-specific timber price lists, and the extent to which the natural regeneration of birch or pine can be exploited. However, an additional issue of importance is how economic considerations are evaluated. For example, greater or lesser emphasis may be placed on the importance of achieving greater yields, versus the importance of minimizing economic or ecological risks (Knoke et al. 2008). Depending on the disturbance of primary concern, mixtures may have a distinct advantage when evaluated from a risk minimization perspective and thus be favored even if the yield is equivalent or even less than monocultures. With respect to such economic risks, the two mixtures considered should also provide owners with increased management flexibility relative to monocultures.

Production forest alternatives must also be evaluated with respect to their capacity to address two problematic challenges posed by anthropogenic climate change: increased uncertainty and risk. Climate change is already affecting the capacity of production forests to deliver ecosystem services, due to altered environmental conditions and increased frequency and the extent of disturbances (Seidl et al. 2014). Over the coming century, the uncertainties inherent to climate change projection and long-term forest management (Millar et al. 2007) will likely be compounded by uncertainties from, for example, the establishment of new pests and pathogens, and the altered behavior and physiology of pest and pathogen species already present within a system (Pautasso et al. 2010). One of the principal recommended strategies for addressing such uncertainties is to “spread the risk” by diversifying tree species composition at stand and landscape scales (Felton et al. 2010a; Pawson et al. 2013). The inclusion of birch or pine in an otherwise spruce-dominated stand can thus be seen as an effective risk-spreading strategy, as it provides owners and managers with alternative directions for stand development when unforeseen disturbance events occur (Millar et al. 2007).

Targeted efforts are also required to reduce the vulnerability of stands to the specific disturbances projected to increase within a region due to anthropogenic climate change. Of direct concern with respect to spruce monocultures is the potential increased risk of pest and pathogen outbreaks, and climatic conditions more conducive to storm damage (Grundmann et al. 2011). Both spruce–birch and spruce–pine mixtures appear to reduce stand vulnerability to such risks. This can be considered to be a win–win adaptation strategy, as the use of these mixtures simultaneously diversifies stand conditions, to address the risks and uncertainties of climate change, while concurrently retaining the tree species for which the most extensive ecological and silvicultural knowledge base exists in Sweden (i.e., spruce).

There is an important and necessary caveat, however, with respect to linking mixture adoption with risk reduction, as spruce–pine mixtures may in fact increase stand vulnerability to some climate-related disturbances. Climate change could bring drier summer climates to southern Sweden and, if coupled with prolonged periods without precipitation, may increase the risk of forest fires (Kjellström et al. 2014). In such cases, the addition of pine to spruce production forests may in fact increase the risk of fire-related production losses (Fig. 1b). This highlights the importance of not conflating the adoption of mixtures with a generic capacity to reduce stand vulnerability to disturbance. Any resultant reductions in risk will be individual to the specific mixture’s tree species composition, regional context, and disturbance type (e.g., wind, fire, pest, and pathogen species) considered. The response of forest owners to recent storm damage in Sweden helps illustrate this point.

Concerns regarding the vulnerability of Sweden’s production forests to climate change rose after a storm hit southern Sweden in 2005 and damaged 75 million m^3^ of wood within what was primarily spruce-dominated forests (Svensson et al. 2011). As a result, compensatory governmental funding was specifically targeted to encourage forest owners to regenerate with broadleaf tree species and associated mixtures. However, due in part to forest owners’ concerns regarding the susceptibility of such stands to damage by browsing ungulates, the majority of this funding went unused (Ulmanen et al. 2012). In this case, both financial incentives and the potential to reduce one long-term risk (windthrow) proved insufficient to overcome the other perceived risks (browsing damage) and uncertainties of adopting mixtures (Lidskog and Sjödin 2014). Whereas financial incentives are often a proposed means of encouraging the adoption of production forest alternatives (Puettmann et al. 2015), the outcomes observed in Sweden indicate how such efforts may readily be derailed if they are inadequate in relation to the perceived risks and uncertainties of the proposed alternative.

## Conclusion

Relative to spruce monocultures, spruce–birch and spruce–pine mixtures appear to provide better outcomes in terms of biodiversity, recreational and esthetic values, water quality, and economic flexibility, as well as addressing some of the growing risks and uncertainties caused by anthropogenic climate change. Despite such benefits, several obstacles to the uptake of these tree species mixtures appear to remain, including browsing pressure, increased management complexity, and a continued uncertainty regarding their economic and production outcomes. On the basis of this study, we hope that research can be targeted toward resolving remaining obstacles and uncertainties, and increased opportunities may be identified for the adoption of mixtures.
